# Thinning of the retinal nerve fiber and choroidal layers in adolescents with anorexia nervosa: A controlled study

**DOI:** 10.5935/0004-2749.2023-0047

**Published:** 2024-03-27

**Authors:** Ceyda Baskan, Alkım Oden Akman, Elif Akcay, Sabite Emine Gökce, Demet Tas

**Affiliations:** 1 Department of Ophthalmology, Ankara Bilkent City Hospital, Ankara, Turkey; 2 Department of General Pediatrics and Division of Adolescent Medicine, Ankara Bilkent City Hospital, University of Health Sciences, Ankara, Turkey; 3 Department of Child and Adolescent Psychiatry, Ankara Bilkent City Hospital, Ankara, Turkey; 4 Department of Ophthalmology, Dr. Abdurrahman Yurtaslan Oncology Research and Training Hospital, Ankara, Turkey; 5 Department of General Pediatrics and Division of Adolescent Medicine, Ankara Bilkent City Hospital, University of Ankara Yıldırım Beyazıt University, Ankara, Turkey

**Keywords:** Anorexia nervosa, Tomography, Optical coherence, Nerve fibers, Choroid, Adolescents

## Abstract

**Purpose:**

We aimed to evaluate retinal nerve fiber and choroidal layer alterations in adolescents with anorexia nervosa using spectral-domain optical coherence tomography.

**Methods:**

Thirty patients with anorexia nervosa and 30 healthy adolescents aged 12-18 years were included in this study. Their age, sex, body mass index, anorexia nervosa type, disease duration, and spectral-domain optical coherence tomography data were recorded.

**Results:**

Central macular thickness and retinal nerve fiber layer thickness in the temporal and inferior regions were significantly lesser in patients with anorexia than in healthy controls (p<0.05). Moreover, significant choroidal thinning around the foveal and subfoveal regions in patients with anorexia was observed (p<0.05). In addition, a statistically significant relation between the increase in disease duration and the thinning of the inferior retinal nerve fiber layer was detected (p<0.05).

**Conclusion:**

The retinal nerve fiber layer and choroidal layer thicknesses were lesser in patients with anorexia than in healthy controls. Screening for retinal indices might prevent the development of irreversible retinal pathologies in adolescents with anorexia nervosa. In addition, thinning of the retinal nerve fiber and choroidal layers could reflect structural or functional changes in the brain of adolescents with anorexia nervosa.

## INTRODUCTION

Anorexia nervosa (AN) is a highly distinct psychiatric disorder that affects individuals of all ages, sexes, sexual orientations, races, and ethnic origins. However, adolescent girls and young adult women are most commonly affected by AN. Patients with AN are intensely fearful of weight gain and have a distorted body image that results in severe dietary restriction or other weight loss behaviors, such as purging or excessive physical activity^(1)^.

According to the Diagnostic and Statistical Manual of Mental Disorders (DSM-V) criteria, AN is an eating disorder characterized by persistent restriction of energy intake. This disorder results in a significantly lower body weight in relation to age, sex, developmental parameters, and physical health^(2)^. AN is a multisystem disease that affects several organs and systems, such as the skin, gastrointestinal system, cardiopulmonary system, hematologic/immunologic system, and hypothalamic--pituitary-ovarian hormonal axis^(3)^.

There are only a few studies that have investigated the ocular complications in AN. These studies describe the anterior segment complications of the eye, including the appearance of corneal ulcers and cataracts, with rod dysfunction in one case and central retinal vein occlusion in another^(4^,^5)^. In another study, optical coherence tomography (OCT) revealed a decrease in the macular and retinal nerve fiber layer (RNFL) thicknesses and multifocal electroretinography revealed a decrease in the electrical activity of the macula in adult patients with AN^(6)^.

Spectral-domain OCT (SD-OCT) with enhanced depth imaging (EDI) is commonly used in clinics for quick and high-resolution analysis of ocular structures. It provides detailed imaging of the retinal and deeper choroidal layers; thus, it has been used to evaluate choroidal changes in several studies^(7^,^8)^. Furthermore, OCT is increasingly being used not only to assess ocular conditions but also to identify central nervous system abnormalities, including psychiatric disorders^(9)^. Because the axons of ganglion cells in the retina are not myelinated, RNFL thinning is considered a marker of neuronal loss^(10)^. Thus, OCT can help us understand the pathophysiology of AN and ocular effects, which are currently poorly understood.

To the best of our knowledge, no study has measured the thickness of the retinal nerve fiber and choroidal layers in adolescents with AN. Here, we aimed to measure the thickness of the macula, RNFL, and choroid using SD-OCT with EDI in adolescents with AN and compare these findings with those from healthy controls.

## METHODS

### Study design and participants

The study population consisted of 30 patients with AN and 30 healthy adolescents that were followed up by the Departments of Child and Adolescent Psychiatry and Adolescent Medicine. The following patient data were obtained from hospital records: height, weight, body mass index (BMI), and medical histories (e.g., eating disorder type, disease duration, and weight change). BMI was calculated by dividing the body weight (kg) by the square of the height (m^2^). The Control Group included healthy children who visited our department for routine examination and matched children in the AN Group in terms of age and sex. Prior to the data collection, Ankara Bilkent City Hospital’s Educational Support Offices and Student Health Services were informed about the study and granted approval to implement the study at each site. Before inclusion in the study, informed consent was given by all participants. Patients with systemic disorders, such as osteoporosis, anemia, cardiovascular complications, rheumatologic disorders, electrolytic abnormalities, and vitamin A and B12 deficiencies, were not included in this study. Patients with a history of ocular surgery, high refractive errors, and retinal problems were also excluded to avoid their effect on OCT findings. All patients underwent a detailed ophthalmological examination, including slit-lamp biomicroscopy; measurement of the visual acuity, refractive error, and intraocular pressure; and dilated fundus examination. The best visual acuity value (as measured using a Snellen chart as decimals and fractions) was recorded for each patient and healthy control.

### OCT

**Figure 1:**
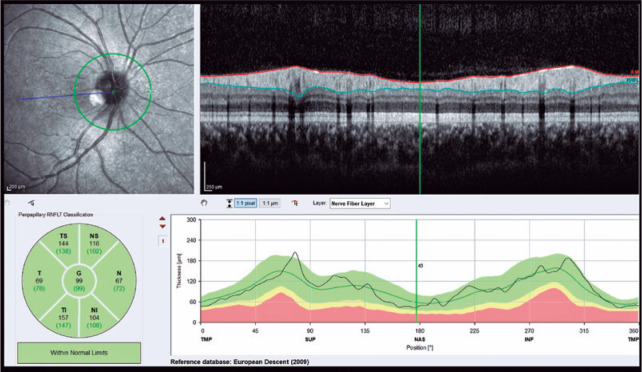
Measurement of the peripapillary retinal nerve fiber layer (RNFL) thickness using spectral-domain optical coherence tomography in a patient with anorexia nervosa (AN). The upper left window depicts the circle scan, which is centered on the optic disc (approximately 3.4 mm in diameter). The top right window shows the RNFL thickness, which was determined by measuring the distance between the internal limiting membrane and the RNFL border. The lower left area displays the classification result for the average of the circle scan (G) and the six standard sectors: temporal (T), temporal superior (TS), temporal inferior (TI), nasal (N), nasal superior (NS), and nasal inferior (NI). The lower right window displays the RNFL thickness profile measured along the circle scan and a comparison between it and the normal range.

**Figure 2:**
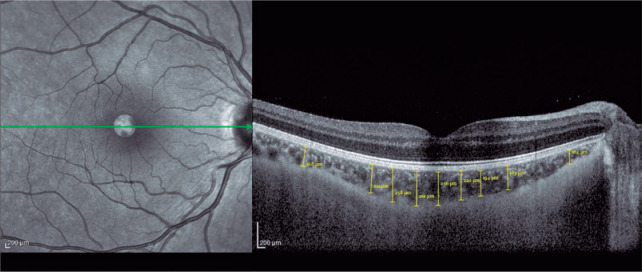
Measurement of choroidal thickness using spectral-domain optical coherence tomography in a patient with anorexia nervosa. The thickness was measured manually at the foveal center and 500, 1000, 1500, and 3000 µm from the foveal center within the nasal, temporal, inferior, and superior quadrants.

**Figure 3:**
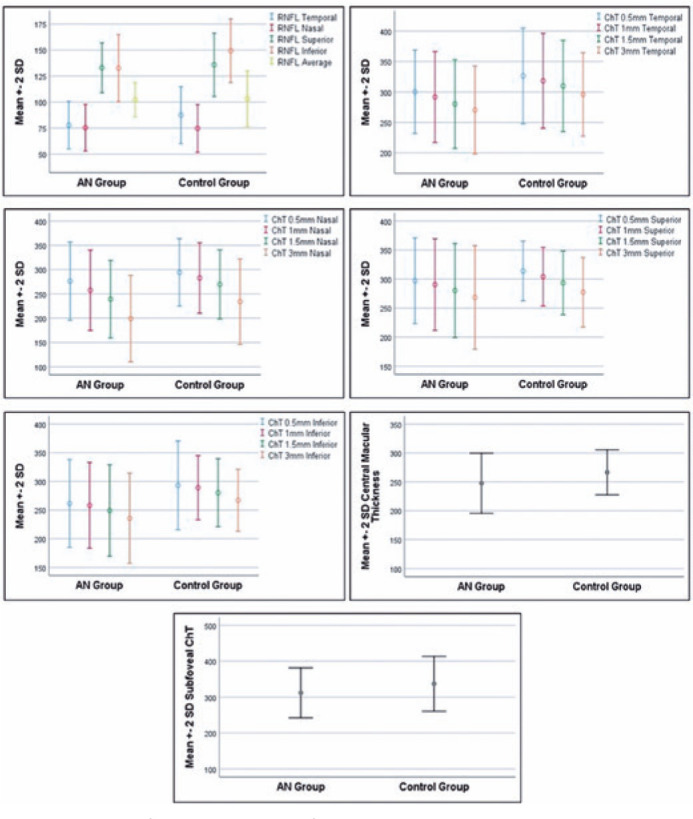
Box plots of the optical coherence tomography measurements of the right eye of both groups. AN, anorexia nervosa; RNFL, retinal nerve fiber layer; ChT, choroidal thickness; SD, standard deviation.

SD-OCT (Spectralis; Heidelberg Engineering, Heidelberg, Germany) was used to visualize the RNFL and choroid. The scan quality ranged from 0 (no signal) to 40 (excellent), and only high-quality images (images with a centered and well-focused optic disc with a signal strength of >20 Db) were selected. The EDI mode was used to improve choroid visualization. Choroidal measurements were performed by two independent experienced physicians who were blinded to the patients. Examinations were performed between 9:00 and 11:00 AM to avoid diurnal variations^(11)^. The right eye values were used for statistical analyses. RNFL thickness was determined using an optic nerve head scan. A volumetric scanning protocol was used to visualize a 15 × 15 region surrounding the optic nerve head (circle scan size: 3.4 mm). The average thicknesses of RNFL and four quadrants (superior, nasal, inferior, and temporal; 90° each) were automatically measured using SD-OCT in the peripapillary area (Figure 1). Choroidal thickness was defined as the distance between the outer retinal pigment epithelial line and the hyperreflective line behind the large choroidal vessel layer at the scleral interface. The thickness was measured manually at 17 different points: at the foveal center and 0.5, 1, 1.5, and 3 mm from the foveal center within the nasal, temporal, inferior, and superior quadrants (Figure 2). Box plots of the OCT measurements for the AN and control groups are shown in figure 3.

### Statistical analysis

All data analyses was performed using SPSS (version 25.0; IBM Corp., Armonk, NY, USA). Data were described using frequency [percentage], mean ± standard deviation, and median with min-max, and chi-square (χ^^2^^) test was used to compare qualitative data. The conformity of the data to normal distribution was evaluated using Kolmogorov-Smirnov and Shapiro-Wilk tests, skewness-kurtosis, and graphical methods (e.g., histogram, Q-Q plot, stem and leaf plot, and boxplot). Independent samples t-test (t-test in independent groups) was used to compare the normally distributed quantitative data between groups. The relationships between the variables were evaluated using Pearson’s correlation test. A p-value of 0.05 was considered statistically significant.

## RESULTS

The study included 30 patients with AN (26 females and 4 males) and 30 healthy adolescents (23 females and 7 males). The average disease duration in the AN Group was 11.1 ± 9.2 months. The mean age in the AN and Control groups was 15.0 ± 1.6 years and 15.3 ± 1.6 years, respectively. The mean BMI in the Control Group was 22.8 ± 1.6. The demographic and disease characteristics of the participants are summarized in table 1. A comparison of the right eye measurements between the two groups is presented in table 2. The temporal (77.8 ± 11.4 µm vs. 87.5 ± 13.7 µm ) and inferior (132.6 ± 16.1 µm vs. 149.4 ± 15.3 µm) RNFL thicknesses were significantly lesser in the AN Group than in the Control Group (p<0.05). The choroidal thickness in all temporal and inferior regions (0.5, 1.0, 1.5, and 3.0 mm from the fovea) was significantly lesser in the AN Group than in the Control Group (p<0.05). The choroidal thicknesses in the nasal regions 1.0, 1.5, and 3.0 mm from the fovea were also statistically lesser in the AN Group than in the Control Group (p<0.05). In the superior region, the choroidal thickness 0.5 mm from the fovea was significantly lesser in the AN Group than in the Control Group (p<0.05). The central macular (247.6 ± 26.0 µm vs. 266.6 ± 19.5 µm ) and subfoveal (311.8 ± 34.7 µm vs. 337.0 ± 38.2 µm) choroidal thicknesses were significantly lesser in the AN Group than in the Control Group (p<0.05). Our analysis did not reveal a significant correlation between any OCT parameter and the patients’ BMI (p>0.05). However, as the disease duration increased, the RNFL thickness in the inferior region significantly decreased (p<0.05). Subgroup analysis revealed no significant difference in any variable between the restrictive and binge--purge AN types. The best-corrected visual acuity was 6/6 in all eyes.

**Table 1:** Demographic and disease characteristics of patients with AN

	Avg. ± SD	Median (Min-Max)
Age (years)	15.1 ± 1.6	15.0 (12.0-17.0)
Weight (kg)	41.0 ± 8.7	39.0 (27.0-66.0)
Height (cm)	158.9 ± 6.0	159.5 (150.0-171.0)
BMI (kg/m^2^)	16.1 ± 2.7	15.9 (11.4-24.0)
Disease duration (months)	11.1 ± 9.2	7.0 (1.5-36.0)
Weight loss	15.6 ± 8.9	13.0 (4.0-45.0)
	N	%
Sex^*^		
Female	26	81.7
Male	4	18.3
Excessive exercise^*^		
Absent	8	26.7
Present	22	73.3
Vomiting^*^		
Absent	24	80.0
Present	6	20.0
Restriction^*^		
Absent	28	93.3
Present	2	6.7
AN type^*^	18	60.0
Binge eating/purging		
Restrictive	10	33.3
Atypical	1	3.3
Post-AN bulimia	1	3.3

* Data are represented as n and %. AN= anorexia nervosa; Avg= average; SD= standard deviation; BMI= body mass index.

**Table 2:** Comparison of retinal and choroidal measurements between anorectic patients and controls

	AN Group (n=30)	Control Group (n=30)	p-value
Sex			
Female	26 (86.7%)	23 (76.7%)	0.505 ^a^
Male	4 (13.3%)	7 (23.3%)	
Age (year)	15.0 ± 1.6	15.3 ± 1.6	0.424 ^b^
RNFL (µm)			
Temporal	77.8 ± 11.4	87.5 ± 13.7	**0.004** ^b^
Nasal	75.4 ± 11.2	74.7 ± 11.5	0.820 ^b^
Superior	132.9 ± 12.0	135.8 ± 15.2	0.410 ^b^
Inferior	132.6 ± 16.1	149.4 ± 15.3	**0.000** ^b^
Average	102.3 ± 8.2	103.3 ± 13.4	0.746 ^b^
ChT (µm)temporal			
0.5 mm	300.3 ± 34.2	326.4 ± 39.3	**0.008** ^b^
1.0 mm	291.7 ± 37.3	318.3 ± 38.9	**0.009** ^b^
1.5 mm	280.3 ± 36.4	309.9 ± 37.5	**0.003** ^b^
3.0 mm	270.5 ± 36.1	296.0 ± 34.3	**0.007** ^b^
ChT (µm) nasal			
0.5 mm	276.2 ± 40.3	294.3 ± 34.7	0.067 ^b^
1.0 mm	257.5 ± 41.4	282.8 ± 36.3	**0.015** ^b^
1.5 mm	239.0 ± 39.9	269.6 ± 35.6	**0.003** ^b^
3.0 mm	199.1 ± 44.5	234.0 ± 43.9	**0.003** ^b^
ChT (µm) superior			
0.5 mm	297.1 ± 36.9	313.8 ± 25.7	**0.046** ^b^
1.0 mm	290.4 ± 39.4	304.1 ± 25.2	0.116 ^b^
1.5 mm	280.3 ± 40.5	293.5 ± 27.4	0.145 ^b^
3.0 mm	268.3 ± 44.6	277.3 ± 29.9	0.361 ^b^
ChT (µm) inferior			
0.5 mm	261.6 ± 38.3	293.2 ± 38.7	**0.002** ^b^
1.0 mm	258.2 ± 37.4	289.0 ± 27.9	**0.001** ^b^
1.5 mm	249.3 ± 39.9	280.3 ± 29.6	**0.001** ^b^
3.0 mm	235.7 ± 39.3	267.1 ± 27.0	**0.001** ^b^
Central macular thickness (µm)	247.6 ± 26.0	266.6 ± 19.5	**0.002** ^b^
Subfoveal ChT (µm)	311.8 ± 34.7	337.0 ± 38.2	**0.010** ^b^

a= Chi-square test (n [%]); b= Independent samples t-test (mean ± SD).

AN= anorexia nervosa; RNFL= retinal nerve fiber layer; ChT= choroidal thickness.

## DISCUSSION

AN is a disease that often begins during adolescence and has long-term irreversible serious complications. It can affect almost every organ system. However, the number of studies on the impact of nutrition on the retina in patients with AN is limited. Thus, we aimed to evaluate RNFL and choroidal layer alterations in patients with AN using SD-OCT and found a significant decrease in their thickness compared with healthy individuals.

Despite reports of affected vision in patients with AN, the underlying pathophysiology of ocular alterations in these patients remains unclear. Dopamine is reportedly associated with AN-related visual issues. It is an important neurotransmitter in the human retina and plays a major role in visual pathways. Dopamine modulates the activity of retinal cells, particularly the photoreceptors and bipolar cells. It affects the way the retina processes visual information and influences the brain’s perception of visual stimuli. Dopamine might also have a protective effect on retinal cells. It has been reported to preserve the health and function of retinal neurons, which are crucial for maintaining vision^(12)^. Furthermore, dopamine regulates eye growth. By controlling the elongation of the eyeball, it can potentially prevent or reduce the progression of myopia^(13)^. Studies on the activity of the dopaminergic system in patients with AN have demonstrated altered brain activity with a reduction in the cerebrospinal fluid concentration of the dopamine metabolite homovanillic acid, which may explain the retinal disturbances^(14)^. Laurence et al. reported that impaired appetitive function in patients with AN leads to disturbances in visual discrimination learning, indirectly indicating altered dopaminergic neurotransmission in patients with AN^(15)^. Parkinson’s disease (PD) provides a better understanding of visual symptoms associated with dopamine deficiency^(16)^. In patients with PD, a reduction in the amplitude of central oscillatory potentials^(16)^ and a decrease in dopaminergic input to a subset of ganglion cells have been observed^(17)^. These changes lead to abnormal production of glutamate and subsequently atrophy and localized thinning of the RNFL^(17)^.

SD-OCT devices generate rapid and high-resolution images that provide three-dimensional visualization of both the RNFL and choroid^(18)^. Furthermore, SD-OCT can effectively detect early changes in the optic nerve before the occurrence of clinically significant long--term damage to the neuronal tissue^(19^,^20)^. Therefore, we used SD-OCT to detect early changes in the RNFL and choroid before the appearance of clinically established ocular pathologies in patients with AN. In the present study, we found that temporal and inferior region RNFL measurements were significantly lower in the AN group than in the control group. The central macular and subfoveal choroidal thicknesses were also lower in the AN group than in the control group. Only a few studies have elicited the ophthalmic findings of AN. Moschos et al. evaluated macular thickness in two different studies^(6^,^21)^. Similar to our findings, they identified a decrease in the central macular thickness in patients with AN. However, they determined that the RNFL thickness did not demonstrate a significant difference between the AN and control groups in most areas, except in the inferior region. In addition to the inferior region RNFL thickness, our results showed a significant decrease in the temporal region RNFL thickness, which has not been previously reported. Temporal RNFL is vulnerable to oxidative stress and energy depletion because it is made up of thinly myelinated, small, parvocellular axons, which might explain our study findings^(22^,^23)^. Moschos et al.^(6)^ also identified a negative correlation between AN duration and the superior, inferior, and average RNFL thicknesses. Similarly, we found a significant decrease in the inferior region RNFL thickness as the disease duration increased. As our study was conducted in the adolescent age group, our results demonstrated that inferior and temporal RNLF thicknesses affected firstly in AN. Moschos et al. did not find any correlation between the OCT parameters and the patients’ BMI^(6)^. Similarly, we did not find any statistically significant relationship between any of the OCT variables and the BMI of patients with AN.

According to the American Psychiatric Association’s DSM-V, there are two types of AN: restrictive and binge--purge. Patients with restrictive AN reduce their daily caloric intake, whereas those with binge-purge AN occasionally experience binging episodes followed by purging behaviors, such as vomiting induction and laxative and diuretic abuse^(2)^. In a previous study with a limited number of patients (six with restrictive AN and seven with binge-purge AN), macular thickness was higher in patients with restrictive AN than in those with binge-purge AN^(6)^. We also performed a subgroup analysis in our patients. However, we did not find a significant difference in the variables between the restrictive and binge-purge AN types. Nevertheless, the large number and relatively uniform distribution of the patients may have allowed us to demonstrate significant temporal RNFL thinning in adolescents with AN, which has not been previously demonstrated.

Circulatory complications in AN have been studied more extensively than neuroendocrine implications of dopamine neurotransmission. AN is reportedly associated with poor peripheral circulation, which can present as cold hands, acrocyanosis, and even Raynaud’s phenomenon^(24^-^26)^. The ocular findings observed in AN may be caused by the decrease in perfusion to the choroid^(27)^. The choroid is an important vascular layer of the globe and plays an important role in the retinal blood supply. Therefore, peripheral hypoperfusion may have decrea-sed the choroidal thickness in our study’s AN group. There have also been reports of regional cerebral blood flow abnormalities in patients with AN, which returns to normal in most cases after weight gain^(28^,^29)^. These anticipated cranial circulatory disturbances might potentially cause choroidal circulation impairment, which may explain the choroid and retinal thickness changes in patients with AN. We found significant choroidal thinning around the foveal and subfoveal regions in the AN Group. Similarly, Moschos et al. found diffusely decrea-sed foveal choroidal thickness in patients with AN^(21)^. These findings are important because the choriocapillaris density further decreases significantly with aging^(30)^. Therefore, age-related modifications in the choroidal vasculature might be more pronounced in patients with AN than the normal healthy population. Decreased RNLF and choroidal thicknesses might indicate the need for the prevention of long-term retinal pathologies that may develop if rehabilitation is not provided.

Our study results indicate that RNFL thinning in adolescents first begins in the inferior and temporal regions. However, further comprehensive studies are required to validate these findings.

The strengths of our study include larger sample size compared with previous studies, evaluation of adolescents, and comparison with a control group. This study also had some limitations. Our follow-up period was short and we were not able to detect the long-term effects of our OCT findings on visual performances in patients with AN. Another limitation was the lack of control measurements after weight gain in patients.

In conclusion, structural changes can occur in the retina and choroid without loss of vision in patients with AN. We determined that AN can affect the retina and choroid even at an early age. Furthermore, patients should be evaluated using OCT, as these deficits are early indicators of visual impairment that can develop later. Moreover, thinning of the RNFL and choroidal layer can reflect the potential structural or functional changes in the brain of adolescent patients with AN. Future studies are required to validate these findings and evaluate adolescents after AN treatment.
